# Mild hypothermia causes differential, time-dependent changes in cytokine expression and gliosis following endothelin-1-induced transient focal cerebral ischemia

**DOI:** 10.1186/1742-2094-8-60

**Published:** 2011-05-31

**Authors:** An-Gaëlle Ceulemans, Tine Zgavc, Ron Kooijman, Said Hachimi-Idrissi, Sophie Sarre, Yvette Michotte

**Affiliations:** 1 Department of Pharmaceutical Chemistry and Drug Analysis, Center for Neuroscience, Vrije Universiteit Brussel, Laarbeeklaan 103, Brussels, 1090, Belgium; 2 Department of Pharmacology, Center for Neuroscience, Vrije Universiteit Brussel, Laarbeeklaan 103, Brussels, 1090, Belgium; 3 Critical Care Department and Cerebral Resuscitation Research Group, Center for Neuroscience, Vrije Universiteit Brussel, Laarbeeklaan 103, Brussels, 1090, Belgium

**Keywords:** Stroke, Hypothermia, Neuroinflammation, Cytokines, Gliosis

## Abstract

**Background:**

Stroke is an important cause of morbidity and mortality and few therapies exist thus far. Mild hypothermia (33°C) is a promising neuroprotective strategy to improve outcome after ischemic stroke. However, its complete mechanism of action has not yet been fully elaborated. This study is the first to investigate whether this neuroprotection occurs through modulation of the neuroinflammatory response after stroke in a time-dependent manner.

**Methods:**

The Endothelin-1 (Et-1) model was used to elicit a transient focal cerebral ischemia in male Wistar rats. In this model, the core and penumbra of the insult are represented by the striatum and the cortex respectively. We assessed the effects of 2 hours of hypothermia, started 20 minutes after Et-1 injection on neurological outcome and infarct volume. Furthermore, pro- and anti-inflammatory cytokine expression was determined using ELISA. Microgliosis and astrogliosis were investigated using CD-68 and GFAP staining respectively. All parameters were determined 8, 24, 72 hours and 1 week after the administration of Et-1.

**Results:**

Et-1 infusion caused neurological deficit and a reproducible infarct size which increased up to 3 days after the insult. Both parameters were significantly reduced by hypothermia. The strongest reduction in infarct volume with hypothermia, at 3 days, corresponded with increased microglial activation. Reducing the brain temperature affected the stroke induced increase in interleukin-1β and tumor necrosis factor α in the striatum, 8 hours after its induction, but not at later time points. Transforming growth factor β increased as a function of time after the Et-1-induced insult and was not influenced by cooling. Hypothermia reduced astrogliosis at 1 and 3 days after stroke onset.

**Conclusions:**

The beneficial effects of hypothermia after stroke on infarct volume and functional outcome coincide with a time-dependent modulation of the cytokine expression and gliosis.

## Background

Stroke is an important cause of morbidity and mortality in industrialized countries and few therapies exist thus far. It is generally acknowledged that many agents, proven neuroprotective in experimental models, fail in clinical practice, possibly because they cannot respond to the complex multifaceted nature of the ischemic cascade after stroke [1-3]. Hypothermia is a well established and robust neuroprotective treatment and has been the focus of research as it may act on several pathways simultaneously [4,5]. The most common form of stroke is a transient focal cerebral ischemia attacking the Middle Cerebral Artery (MCA) or one of its collaterals [1]. In the endothelin-1 (Et-1) rat model, a potent vasoconstrictor is infused in the vicinity of the MCA, thereby occluding this vessel (>75%) during 30 minutes before gradual reperfusion takes place [6]. The core and the penumbra of the insult are represented by the ipsilateral striatum and cortex, respectively. In this and in other MCA occlusion (MCAO) models, mild hypothermia (33°C) has proven to reduce the infarct volume by half, 24 hours after the administration of Et-1, as the result of an almost complete recovery of the penumbra [7,8]. We also showed that in the Et-1 model, reduction in infarct size by hypothermic treatment coincides with decreased levels of apoptosis and oxidative stress in the penumbra [9]. However, the influence of hypothermia on neuroinflammation and neurological outcome has not yet been investigated in this model. Shortly after stroke, cytokines are released from a variety of cells in the central nervous system. The most important ones produced are interleukin-1 beta (IL-1β) and tumor necrosis factor alpha (TNF-α), the former described as a pro-inflammatory cytokine and the latter as a more pleiotropic one with neurotoxic as well as neuroprotective properties [10-12]. Transforming Growth Factor beta (TGF-β) on the other hand is an anti-inflammatory cytokine that is also upregulated after stroke and serves to protect the brain against ischemic injury [13]. The primary source of IL-1β is activated microglia or macrophages, followed by astrocytes. Some production exists from neurons, endothelial cells, oligodendroglia and circulating immune cells [14]. TNF-α and TGF-β, in general, follow IL-1β's production profile [15,16]. Consequently, an essential role in the upregulation of IL-1β, TNF-α and TGF-β can be assigned to activated microglia and macrophages [17,18]. Higher concentrations of these cytokines further activate microglia and astrocytes and a full-blown inflammatory response develops, usually leading to an aggravation of the damage after stroke [16,19]. It is generally accepted that hypothermia is able to reduce the inflammatory response after stroke [4,20]. However, there is no evidence whether this reduction occurs in the core of the insult and/or in the penumbra. In addition, short-term effects of treatment may be different from long-term effects. Indeed, recent literature indicates that cerebral ischemia induces a biphasic response including a quick, merely detrimental, inflammatory response and a slow response, which could be beneficial. Moreover, the switch depends on the severity of the insult. For example, at an early stage, reactive microglia have shown neurotoxic properties, while sustained activation appeared beneficial to neuronal repair [16,21]. To address these essential questions, we investigated the effects of a 2 hours mild hypothermic treatment on microgliosis, astrogliosis and cytokine production as a function of time up to 1 week after an Et-1-induced insult and compared the results with neurological outcome and infarct size at the same time points. This study is the first to show that hypothermia has a modulatory effect on the neuroinflammatory response after an Et-1-induced stroke.

## Methods

The experiments were performed according to the National Guidelines on Animal Experimentation and approved by the Ethical Committee for Animal Experimentation of the Faculty of Medicine and Pharmacy of the Vrije Universiteit Brussel.

### Surgical Procedure and the induction of stroke

Male albino Wistar rats (Charles River Laboratories, IFFA-CREDO, Germany) weighing between 270-300 g were treated as described previously by Van Hemelrijck *et al*. (2005). Briefly, 24 hours before the induction of the insult, the animals were anaesthetised with a mixture of ketamine/diazepam (50/5 mg/kg i.p.) for implantation of 2 intracerebral guides according to the atlas of Paxinos & Watson [22]. One was positioned in the pyriform cortex, close to the MCA (coordinates relative to bregma AP +0.9 mm, L +5.0 mm and V +2.8 mm), the other one in the contralateral prefrontal cortex (AP +3.2 mm, L -3.0 mm, V +2.3 mm). Immediately after surgery, rats received 4 mg/kg ketoprofen i.p. The next day, rats were anaesthetised with 4% sevoflurane (Sevorane^®^, Abbott, Kent, England) and oxygen, insufflated into a transparent chamber and were maintained under this inhalation anaesthesia using 1.5% sevoflurane delivered with oxygen at 0.8 L/min via a facemask throughout the experiment. The first guide was replaced by a cannula (CMA, 3 mm probe with removed membrane, Stockholm, Sweden) by which 500 pmol Et-1 (Sigma, St-Louis, MO, USA), dissolved in Ringer's solution, at a rate of 1 μl/min during 6 minutes, was infused. The second guide was substituted by a hypodermic needle probe (HYP-O-SLE, Omega Corporation, Stamford, USA), adjusted to measure the brain temperature continuously in the contralateral hemisphere. These temperature probes are accurate up to 0.5°C.

### Experimental groups

Hypothermia was started 20 minutes after initiation of Et-1 infusion. In 10 minutes, brain temperature was reduced to 33.0 ± 0.5°C by spraying alcohol onto the animal and cooling it with a fan to target temperature. This reduced temperature was maintained for 2 hours, after which the animals were gradually rewarmed during 30 minutes. Subsequently, the rats were kept at 37°C for another 30 minutes before anaesthesia was stopped. Hypothermic animals were always compared to normothermic rats in which the brain temperature was kept constant at 37.0 ± 0.5°C throughout the experiment [23]. A normothermic sham group was included in which Ringer's solution was infused (hereafter called sham rats). Rats were sacrificed at 8, 24, 72 or 168 hours (1 week) after the administration of Et-1. In total, 137 animals were used for this study. Infarct size and gliosis were assessed in the same rats (n = 76), while cytokine levels were measured in different animals (n = 61).

### Neurological deficit score (NDS)

Sensorimotoric outcome after stroke was assessed using a NDS as described in Garcia *et al*. (1995). Six small tests were performed and scored on the animal in order to estimate the degree of neurological deficit after the induction of the insult. This score resembles the NIHSS score used in clinical practice closely. Firstly, spontaneous activity was determined, followed by symmetry in the movement of the limbs, forepaw outstretching and climbing. These last 3 parameters assessed differences in the use of the contralateral forepaw after inducing the insult. Finally, the equality in response to touch was determined in the last 2 parameters: response to body and vibrissae touch. By scoring all these different tests, a score was given between 3 (the worst) and 18 (the best) [24]. These parameters were assessed before and 24, 72 and 168 hours after the insult. Surgery had no effect on the NDS (data not shown).

### Infarct size

Rats were sacrificed with 6% sodium pentobarbital and transcardially perfused with saline followed by freshly prepared buffered 4% paraformaldehyde (250 ml in 5 minutes). The brains were dissected out and post-fixed in the same buffer. Fifty μm thick slices were cut and preserved on phosphate buffered saline (PBS, 0.01 M) with sodium azide (0.1%) as a preservative. To quantify the infarct volume stereologically, a Nissl staining was performed on these slices every 200 μm, when mounted onto gelatine coated glasses. Pictures of the slices at a magnification of x1.25 were transferred to a computer. After scaling, the marked infarcted area in mm^2 ^was calculated using Image J (NIH, version 1.37). The infarct volume in mm^3 ^was determined by multiplying these values with the interspace. A correction for edema was made according to the following formula: infarct area × (area contralateral hemisphere/area ipsilateral hemisphere) [25].

### Immunohistochemistry (IHC)

Phagocytic cells, originated from microglial activation and macrophage infiltration, can be defined by CD-68 expression. Astrogliosis was measured using glial fibrillary acidic protein (GFAP) expression. Both were determined by IHC on 50 μm thick slices, obtained as described above. In short, after pre-incubation with 0.01% Triton X-100, 3% hydrogen peroxide and pre-immunized goat serum (1:5 dilution, Sigma, St-Louis, MO, USA), brain slices were incubated overnight at 4°C, with polyclonal mouse anti-CD-68 (1/1000 in PIG/PBS 1/5, MCA341R, AdB Serotec, Düsseldorf, Germany) or polyclonal rabbit anti-GFAP (1/10000 in PBS, Z0334, DakoCytomation, Glostrup, Denmark). Next, the slices were incubated for 4 hours at room temperature with a 1/100 dilution of either sheep anti-mouse or monkey anti-rabbit secondary antibody (NA931V and NA934V respectively, Amersham, GE Healthcare, Buckinghamshire, UK). Antibody binding was visualized using the diaminobenzidine substrate chromogen kit (DakoCytomation, Glostrup, Denmark). Between all incubations, a washing step was performed with PBS/0.1% Tween-20.

For each rat, IHC protocols were performed on 3 slices taken from 0.2 mm anterior to bregma to 0.26 mm posterior to bregma [22]. In this region, interference of the insertion of the probe to inject Et-1 was avoided. To compare GFAP expression in sham, normothermic and hypothermic rats, the relative GFAP staining intensity was calculated in each rat: (mean gray value ipsilateral striatum (or cortex) - mean gray value contralateral striatum (or cortex)), using Image J (NIH, version 1.37) [26,27]. Calculating the difference in intensity between the ipsi- and the contralateral side is advantageous to correct for variations in circumstances when the IHC protocols were performed. Phagocytic cells were counted on several fields of 6 mm^2 ^area (magnification x10). By using reference lines, perpendicular to each other, a lattice containing 1 mm^2 ^squares was placed in the same way over every brain slice. By this technique, orientation in the slice became possible and 3 fields consisting of 6 squares in the striatum and cortex were counted in every brain slice at the same place. The fields were chosen in such a way that the 3 representative parts of both the striatum and cortex were covered. The cells in these fields were counted by 3 independent observers blinded to the experimental design [28].

### ELISA

To measure IL-1β, TNF-α and TGF-β1 protein levels in brain homogenates, a different set of rats was sacrificed following the same experimental protocol. As the core and the penumbra of the insult are represented by the striatum and cortex respectively, these structures were used to determine the levels of the cytokines after stroke. Contra- and ipsilateral striatum and cortex were quickly removed, accurately weighed, snapfrozen and stored at -80°C. Next, tissues were homogenized for 30 seconds in HEPES buffer (pH 7.4) with 2% Protease Inhibitor cocktail (Sigma, St-Louis, MO, USA) and subsequently sonicated for 40 seconds (Branson Sonifier 250). After centrifugation for 30 minutes at 6720 g at 4°C (Sorvall RC5B refrigerated superspeed centrifuge, Dupont Instruments), supernatants were collected and stored at -20°C until use. The concentrations of IL-1β, TNF-α and TGF-β1 were measured using the Quantikine^® ^ELISA kits (R&D Systems, Abingdon, UK), according to the manufacturer's instructions. The kits were originally designed to determine these cytokines in rat serum, but were optimized for brain homogenates. Only for TGF-β1, it was necessary to activate the latent form before detection was possible. This implied incubation with 1M HCl, followed by neutralization of the samples with 1.2M NaOH/0.5M HEPES buffer. The protein content was determined using the Lowry method (Bio-Rad laboratories, California, United States).

### Statistical analysis

All data are expressed as mean ± SEM. Data analysis was performed using the statistical program Graphpad InStat (version 3.06 Windows XP, GraphPad Software, San Diego, California, USA). For statistical testing of differences as a function of time, a one-way ANOVA was used with Dunnett post-hoc test compared to basal values or the ones obtained at 8 hours. At each time point, a one-way ANOVA with Dunnett post-hoc test was used to compare sham and hypothermic rats to the normothermic ones. For statistical testing of the infarct volume and the results of the IHC when corrected for infarct volume, where only 2 groups needed to be compared, an unpaired t-test was used. The level for significance was 0.05 for all statistical testing.

## Results

### Mild hypothermia reduces infarct size and improves neurological outcome after stroke

**Figure 1:**
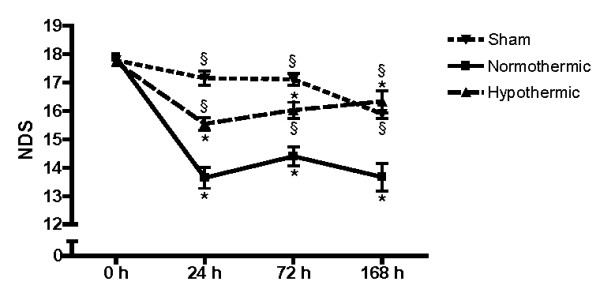
**Evaluation of NDS post-stroke in time**. Effect of hypothermic treatment on the Neurological Deficit Score (NDS) as a function of time in sham (n = 9 at 24 and 72 hours and n = 5 at 168 hours), normothermic (n = 12 at 24 and 72 hours and n = 7 at 168 hours) and hypothermic (n = 14 at 24 and 72 hours and n = 8 at 168 hours) animals. *Significantly different (p < 0.05) from 0 hours as determined by one-way ANOVA followed by Dunnett post-hoc test. ^§^Significantly different (p < 0.05) from normothermic rats as determined by one-way ANOVA followed by Dunnett post-hoc test.

The NDS of operated animals before administration of Et-1 or vehicle was 17.8 ± 0.1 (mean ± SEM, n = 29). Sham animals showed no significant changes in NDS up to 1 week after Ringer's infusion. Induction by Et-1 of focal cerebral ischemia induced a significant reduction in NDS in normothermic animals. The hypothermic treatment significantly improved neurological outcome at all time points studied (Figure 1).

**Figure 2:**
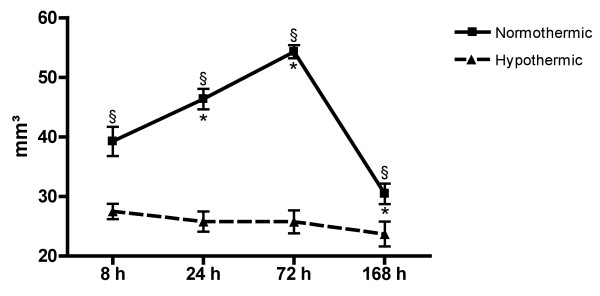
**Hypothermia attenuates the Et-1-induced infarct size up to 1 week**. Effect of hypothermia on the Et-1-induced infarct volume as a function of time in normothermic (n = 4, 10, 5 and 7 at 8, 24, 72 and 168 hours respectively) and hypothermic (n = 5, 8, 7 and 8 at 8, 24, 72 and 168 hours respectively) animals. Data are shown as mm^3 ^(mean ± SEM). *Significantly different compared to the infarct volume at 8 hours as determined by one-way ANOVA with Dunnett post-hoc test. ^§^Significantly different (p < 0.05) from normothermic rats as determined by the unpaired t-test.

Normothermic rats showed a large infarct volume of 39.3 ± 2.5 mm^3^, already at 8 hours after the insult which increased further up to 54.3 ± 1.1 mm^3 ^at 72 hours. Between 72 hours and 1 week, the infarct size decreased by 56% to 30.5 ± 1.7 mm^3^. The hypothermic treatment already prevented the increase in infarct size after 8 hours. Consistent with our previous findings, hypothermia significantly reduced the infarct size by almost half (44.4%) at 24 hours, thereby mostly saving the penumbral area of the infarct [9]. Even at 72 hours, the greater part of the cortex could be salvaged by the hypothermic treatment, resulting in a reduction of the infarct size of 53.4% (Figure 2).

### Mild hypothermia strongly affects microgliosis and astrogliosis

**Figure 3:**
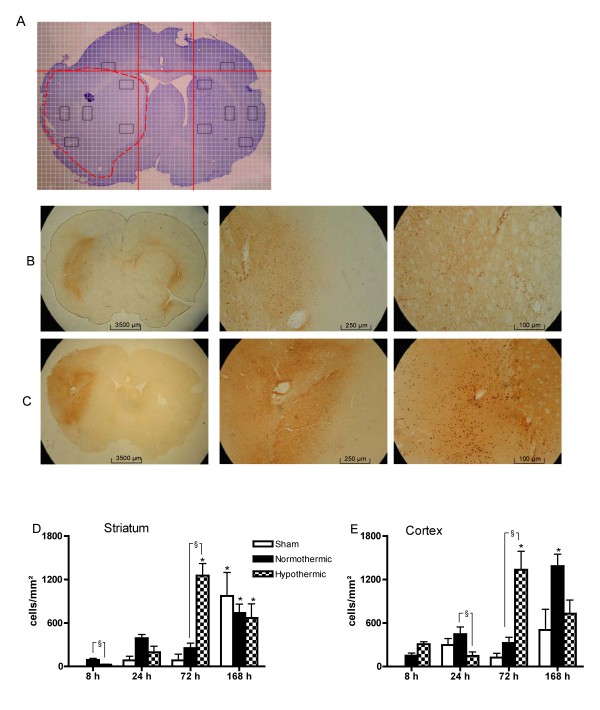
**Hypothermia causes time-dependent changes on CD-68 expression after stroke**. (A) shows the 3 fixed fields in the striatum and the cortex in which CD-68^+^-cells were counted using a lattice. The reference lines are represented in red. The phagocytic cells determined by expression of CD-68 in a normothermic and hypothermic rat at 72 hours after the administration of Et-1 or Ringer's are shown in (B) and (C) respectively. The graphs below show the overall expression in the striatum (D) and the cortex (E) separately. Data are expressed as the number of cells/mm^2 ^and are shown as the mean ± SEM. There was no binding of the second antibody in the absence of the primary antibody (data not shown). *Significantly different (p < 0.05) compared to the results at 8 hours as determined by one-way ANOVA with Dunnett post-hoc test. Furthermore, ^§^normothermic rats (n = 4, 8, 5 and 8 at 8, 24, 72 and 168 hours respectively) were compared to sham rats (n = 4 at 8, 24 and 72 hours and n = 5 at 168 hours) and hypothermic rats (n = 5, 6, 6 and 8 rats at 8, 24, 72 and 168 hours respectively) using one-way ANOVA with Dunnett post-hoc test.

When microglia become activated after brain injury, they alter their immune phenotype and transform into resident macrophages, thereby allowing selective staining by anti-CD-68 (Figure 3) [16]. The way CD-68^+^-cells were counted, is illustrated in Figure 3A. Untreated animals (without surgery) showed no CD-68^+^-cells (n = 4, data not shown). In the brain slices of Et-1-treated rats, there were no positive cells contralateral to the site of the Et-1 injection (data not shown). In sham animals, CD-68 expression remained low. However, 1 week after the insult, an increase was observed in the striatum, possibly due to the longer implantation of the probes. In normothermic conditions, Et-1 administration induced an increase in CD-68 expression both in the striatum and the cortex peaking after 1 week. The hypothermic treatment influenced CD-68 expression differently depending on the time after the insult. At 24 hours, hypothermia attenuated CD-68^+^-cells in the cortex. However, 72 hours after the insult, hypothermic treatment significantly enhanced CD-68 expression about 5-fold compared to normothermic animals in both striatum and cortex.

**Figure 4:**
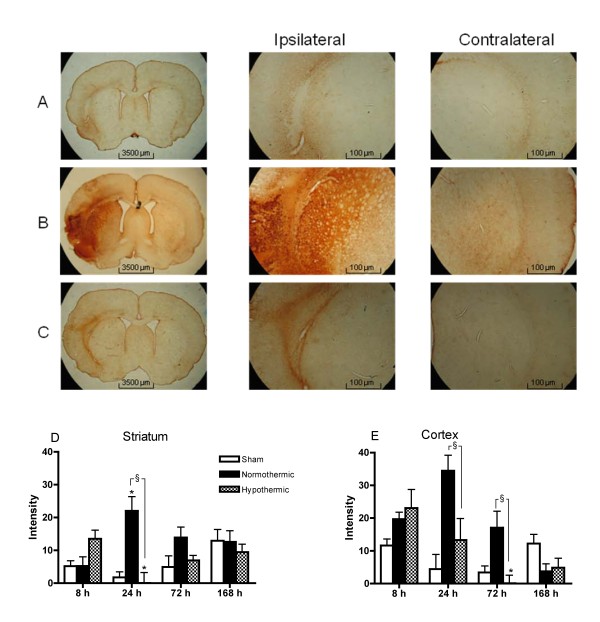
**Hypothermia reduces GFAP staining 24 hours after an Et-1-induced insult**. Activation of astrocytes as determined by the expression of GFAP in a sham (A), normothermic (B) and hypothermic (C) rat, 24 hours after the administration of Et-1 or Ringer's solution. The graphs below show the overall expression in striatum (D) and cortex (E) separately in sham (n = 4 at 8, 24 and 72 hours and n = 5 at 168 hours), normothermic (n = 4, 6, 5 and 6 at 8, 24, 72 and 168 hours respectively) and hypothermic (n = 5, 6, 7 and 8 at 8, 24, 72 and 168 hours respectively) rats. *Significantly different (p < 0.05) compared to 8 hours as determined by one-way ANOVA with Dunnett post-hoc test. ^§^Significantly different (p < 0.05) from normothermic rats as determined by one-way ANOVA with Dunnett post-hoc test.

Untreated animals displayed equal levels of GFAP expression in both hemispheres (data not shown). GFAP expression in the contralateral hemisphere was not affected by any treatment. As observed for CD-68, low expression of GFAP was present in the ipsilateral hemisphere of sham animals. In the normothermic animals, GFAP expression in the striatum peaked at 24 hours after the insult, followed by a gradual decline. Hypothermia significantly suppressed the expression of GFAP in the striatum at 24 hours and in the cortex at 24 and 72 hours after the insult (Figure 4).

**Figure 5:**
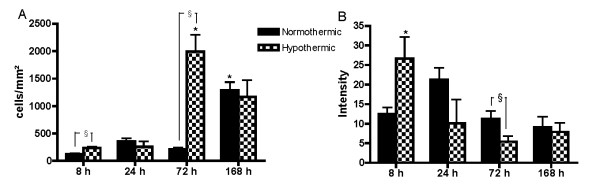
**Microgliosis and astrogliosis corrected for the infarct volume**. Graphs (A) and (B) represent the data of the CD-68 and GFAP staining respectively, when corrected for the infarcted area. All infarct volumes were expressed as a percentage of the infarct size in normothermic rats at 8 hours after the infusion of Et-1. The data show that the observed changes in microgliosis and astrogliosis in this study cannot be solely ascribed to differences in infarcted area. *Significantly different (p < 0.05) compared to 8 hours as determined by one-way ANOVA with Dunnett post-hoc test. ^§^Significantly different (p < 0.05) from normothermic rats as determined by the unpaired t-test.

Correction of the CD-68 and GFAP staining data for infarct size shows that some of the observed effects of hypothermia in the striatum or cortex are modulated by the infarct size, especially at early time points after the insult. Indeed, CD-68^+^-cells which were decreased or increased by the hypothermic treatment at 8 hours in the striatum or cortex respectively, show an overall increase in microglial activation. At 24 hours after the insult, the hypothermia-induced reduction in CD-68^+^-cells is no longer observed after correction of the infarct size. On the other hand, the marked increase in CD-68 expression at 72 hours after hypothermia is even stronger after correction (Figure 5A). In contrast, the decrease in GFAP expression by the hypothermic treatment at 24 and 72 hours is reduced after correction. However, the attenuation at 72 hours is still significant, indicating that the reduction in GFAP expression is in part independent of the reduction in infarct size (Figure 5B).

### Mild hypothermia affects the early production of pro-inflammatory cytokines in the ipsilateral striatum and cortex

**Figure 6:**
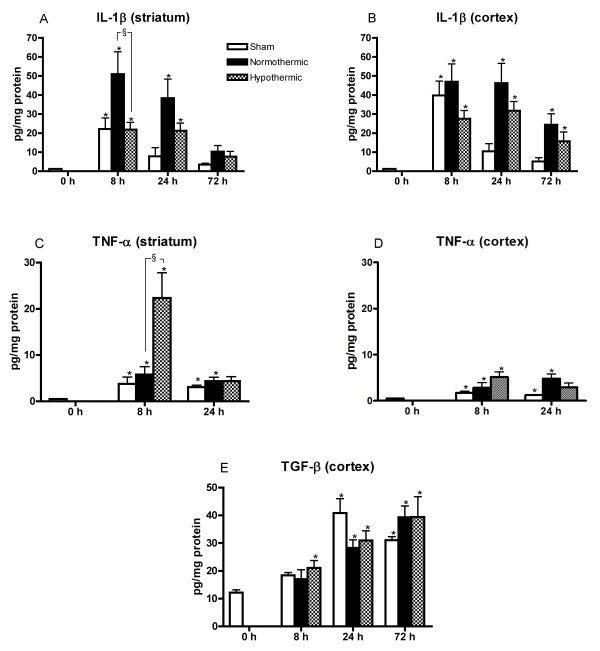
**Determination of cytokine expression after stroke in time and the effect of hypothermia**. Effect of hypothermic treatment on the protein levels of cytokines as a function of time after Et-1 administration for sham, normothermic and hypothermic rats. Levels are expressed as pg/mg protein and are shown as mean ± SEM. Graphs (A) and (B) show the levels of IL-1β at several time points after the administration of Et-1 for striatum and cortex respectively, (C) and (D) for TNF-α and (E) represent the levels of TGF-β in the cortex. The number of rats used to determine the levels for IL-1β and TGF-β were in the sham group: 6, 4 and 4 at 8, 24 and 72 hours; in the normothermic group: 6 at 8, 24 and 72 hours; and in the hypothermic group: 6, 6 and 5 at 8, 24 and 72 hours. The number of rats used to determine the levels for TNF-α was in the sham group: 6 at 8 and 24 hours; in the normothermic group: 5 and 6 at 8 and 24 hours; and in the hypothermic group: 5 and 6 at 8 and 24 hours. Basal levels were measured in rats that underwent no treatment (n = 10) *Significantly different (p < 0.05) from basal levels determined by one-way ANOVA with Dunnett post-hoc test. ^§^Significantly different (p < 0.05) from normothermic rats as determined by one-way ANOVA with Dunnett post-hoc test.

Basal levels of IL-1β and TNF-α were not detectable. Therefore, at 0 hours, their level was equalled to the detection limit (IL-1β: 11.5 pg/ml, TNF-α: 4.7 pg/ml) and divided by the protein content of untreated animals, in order to perform statistical analysis (Figure 6).

IL-1β levels were significantly increased in the sham rats, 8 hours after the infusion of Ringer's solution, both in the striatum and the cortex. Afterwards they decreased back to basal levels (Striatum: 1.2 ± 0.1 pg/mg protein, Cortex: 1.3 ± 0.04 pg/mg protein, n = 10). A similar profile was observed after Et-1 administration in normothermic rats. IL-1β levels were significantly higher than those in the sham animals except at 8 hours in the cortex. One week after the insult, IL-1β was no longer detectable in all treatment protocols. Hypothermia attenuated the IL-1β increase at all time points studied. However, a statistical significant difference was only observed at the 8 hour time point in the ipsilateral striatum.

In all treatment protocols, TNF-α was only detected up to 24 hours after the insult. Furthermore, TNF-α levels were significantly lower in the cortex compared to the striatum (Wilcoxon-matched pairs test, p < 0.05), which is consistent with literature findings [11]. TNF-α levels were slightly increased in sham and normothermic rats until 24 hours after the administration of Ringer's solution compared to basal levels (Striatum: 0.5 ± 0.01 pg/mg protein, Cortex: 0.5 ± 0.02 pg/mg protein, n = 10). Hypothermia significantly enhanced TNF-α levels immediately after the insult in the striatum. In the cortex, this increase did not reach statistical significance. At the 24 hours time point, hypothermia had no significant effect on the Et-1-induced TNF-α levels compared to normothermic animals.

TGF-β1 was only determined in the cortex as the small amount of sample of the striatum hampered its measurement in this structure. Besides, histology showed that hypothermia primarily salvages the penumbra (cortex) [9]. Basal levels of TGF-β1 were detectable: 12.1 ± 1.0 pg/mg protein (n = 10). A gradual increase in TGF-β1 was observed up to 72 hours after the insult. There was no significant difference observed between sham, normothermic or hypothermic rats. Thus, hypothermia had no effect on the possible beneficial effects of TGF-β1 in the cortex.

## Discussion

It is generally accepted that hypothermia improves neurological outcome and reduces inflammation in animal models of focal cerebral ischemia, but no elaborate studies exist in the Et-1 model [7,20,28,29]. It is, however, important to investigate inflammation after stroke in various animal models as the type of model used to induce cerebral ischemia will partially influence the inflammatory response after stroke. The Et-1 model is a good alternative for several reasons. First, to induce the insult, there is no damage to the cerebral vasculature. Secondly, the reperfusion after stroke is gradual which is most common in a clinical situation. Finally, in the Et-1 model, mechanical manipulations are minimized which is ideal for investigating inflammation [25,30]. Furthermore, there is need for additional studies looking at the effects of hypothermia on neuroinflammation at different time points after the insult. The latter is especially relevant as treatments may exert different effects on early versus late inflammatory responses [21]. In this study, the effects of 2 hours of mild hypothermia on neuroinflammation at different time points up to 1 week after the insult were investigated.

A reduction of infarct size by application of mild hypothermia in the Et-1 model has been reported before [9]. Here we show for the first time that this reduction coincides with an improvement in neurological outcome which is sustained for at least one week. Our study confirms the general notion that 2 hours of mild hypothermia, started 20 minutes after stroke onset, exerts anti-inflammatory effects [28,29]. This is reflected by our observation that hypothermia impairs IL-1β production in the early phases after the insult. However, this study is the first to show that hypothermia can enhance CD-68 expression in the striatum and cortex at later time points and augment the expression of TNF-α in the striatum. In the following sections, we will discuss the role of neuroinflammation in the neuroprotective effects of hypothermia.

### The neuroinflammatory response in the Et-1 model

As in other MCAO models, we observed the largest infarct volume at 3 days after the insult, followed by a significant decrease in infarct size at 1 week. This suggests that regions with no living cells (Nissl-negative) at 3 days after stroke onset (especially the core of the insult) become infiltrated by resident and/or blood-borne macrophages after 1 week [25]. Indeed, these cells clear up the necrotic cell debris and may therefore be responsible for the smaller measured infarcted area. Our observation that CD-68 expression peaks at 1 week after the administration of Et-1, supports this hypothesis. Our data are in line with those of Zhang *et al*. (1997) who also observed an increase in this microglial activation up to 166 hours after a 2-hour MCAO [31]. The phagocytic activity of macrophages in the necrotic tissue is considered to be potentially protective [25,31]. Our results are consistent with this notion, as we observed a reduced infarct volume at 1 week after administration of Et-1 which coincides with peak levels of CD-68^+^-cells. Moreover, the neuroprotective influence of activated microglia is suggested by the strong reduction in infarct volume with hypothermia at 3 days, which corresponded with increased levels of CD-68 expression. Neuroprotective effects of microglia have been demonstrated in a mouse model for transient MCAO by selective ablation of proliferating microglia [32]. Furthermore, in a recent study by Narantuya *et al*. (2010), intravenous administration of human microglial cells after a 90 minutes MCAO in rats, improved neurological outcome and reduced infarct size [33]. However, microglia may also exert detrimental effects via the production of inflammatory cytokines. For instance, IL-1β is generally accepted to be a pro-inflammatory cytokine that will enhance cell damage, whereas TNF-α is considered to be a neuromodulator which may exert protective as well as detrimental effects [10-12,16]. Protective effects may be the result of induction of neutrophil apoptosis and their engulfment by macrophages, an effect which seems beneficial as this phagocytosis prevents the lytic release of cytotoxic contents into the surrounding tissue [25,34]. Alternatively, TNF-α may protect neurons from apoptosis through activation of NF-κB [11,35]. In a permanent MCAO mouse model, Legos *et al*. (2000) suggested that production of IL-1β shows a biphasic release pattern with a first peak only a few minutes to 1 hour after stroke onset with a return to basal values at 8 hours and a second peak from 12 hours to 3 days [36]. In our study, we did not observe a biphasic release pattern for IL-1β. These differences could be due to the different models used to study the temporal profile of IL-1β after stroke. TGF-β1, an overall anti-inflammatory cytokine, is upregulated after stroke. It controls proliferation, differentiation, apoptosis and migration of varying cells, like neurons and astrocytes [37]. In contrast to IHC, ELISA on brain homogenates allows easy detection of the latent form of TGF-β1 [13]. We observed a consistent increase of TGF-β1 as a function of time. These results confirm literature findings, which show that TGF-β1 increases up to 1 week after the insult in rats and mice [17,37]. 

Astrocytes are essential elements of the blood brain barrier, they maintain extracellular homeostasis and produce trophic factors [38]. Nowicka *et al*. (2008) described the temporal profile of astrogliosis after stroke in the photothrombosis model. It started with a massive astroglial response in the core of the lesion 4 hours after the trauma and activation was observed up to 28 days after stroke onset [39]. Our results are similar with massive astrogliosis peaking at 1 day. Afterwards, it declines again, but stays visible up to 1 week. Somewhat different are the results from a study by Zhu *et al*. (2000) in a transient forebrain ischemia model (with 10 minutes occlusion) which showed slight activity 1 day after the insult and peak activity at 4 days. However these results were obtained from brain slices from the hippocampus and not from the striatum [37].

### Two hours of mild hypothermia affects the neuroinflammatory response observed after the administration of Et-1

Mild hypothermia improved functional and neurological outcome up to 1 week after stroke. It was important to consider the evolution of both functional recovery and brain tissue salvation, to see if a short hypothermic period can reduce damage long-term after an insult [7,40-42]. In this study, the infarct volume in hypothermic rats never reached the volume observed in normothermic rats at 8 hours. Furthermore, the increase in infarct volume in normothermic animals between 8 and 72 hours was also absent in hypothermic rats. In addition, the improvement in neurological outcome at 24 hours was sustained for at least 6 days and even slightly increased.

The inhibition of IL-1β expression confirms that hypothermia reduces inflammation [7]. In contrast to IL-1β, TNF-α was increased in the ipsilateral striatum after application of hypothermia at 8 hours after the insult. Indeed, at that time point, CD-68 expression in the striatum was decreased, while GFAP levels were increased more than 2-fold in the striatum of hypothermic rats, although not significantly (p = 0.07). It is also possible that hypothermia exerts differential effects on individual genes. This implies that certain genes, such as those for TNF-α, are more strongly stimulated than other genes (e.g. GFAP). Since inhibition of TNF-α activity in a murine MCAO model decreased infarct size, it could be that the increase in TNF-α expression exerts negative effects in our model [43]. Therefore, we have to consider the possibility that the effect of hypothermia is a cumulative outcome of negative and positive effects. However, the influence of TNF-α strongly depends on the relative expression of type I and type II TNF receptors (TNFR1 and TNFR2, respectively) which trigger different signal transduction pathways and cellular effects. For example, a null mutation of TNFR1 in a murine MCAO model resulted in increased neuronal damage compared to controls and mice with a TNFR2 null mutation [35]. Interestingly, Lotocki *et al*. (2006) found that after traumatic brain injury, TNFR1 is highly upregulated after 15 minutes and hypothermia is able to reduce this overexpression so soon after the injury [44].

Whereas the results on CD-68 expression, when corrected for infarct volume, suggest that the inhibitory effect of hypothermia observed in the cortex at 1 day after the insult, is due to reduced infarct volume, the increase in phagocytic cells at 3 days is not. Indeed, CD-68 expression is significantly increased in hypothermic rats at 3 days after the Et-1-induced stroke. Other groups have shown that after 3 days, macrophages phagocytose necrotic cell debris and facilitate plasticity [21,25]. Therefore, the increase in CD-68^+^-cells at 3 days could point to a beneficial increase in macrophages. However, Wang *et al*. (2002) observed a significant decrease in CD-68 expression with hypothermic treatment 3 days after the induction of a 2 hours lasting ischemia. The differences in occlusion time (2 hours versus 30 minutes) and onset of the hypothermic treatment (immediately versus a 20 minutes delay) could explain this discrepancy [20,28]. Furthermore, our observation that the hypothermic treatment still significantly increases the level of CD-68 staining at 3 days after the insult may also explain why delayed hypothermia can still be effective [45-47]. Moreover, the bidirectional data on CD-68 expression, when striatum and cortex are analyzed individually, may be explained by assuming that hypothermia exerts a short- and long-term effect on microglial activation. The short-term effect would consist of a reduction in infarct size and a concomitant decrease in microglial activation in the striatum, while the long-term effect comprises an increase in CD-68 expression at 3 days after the administration of Et-1, possibly as a result of enhanced TNF-α production. Alternatively, because CD-68 is also present on macrophages, the long-term effect (observed at 3 days after the insult) may also be due to increased expression of chemokines leading to enhanced infiltration of macrophages from the periphery.

Astrogliosis is reduced in hypothermic animals 1 and 3 days after the administration of Et-1. Although the changes in GFAP expression after application of hypothermia seem partially dependent on the reduced infarct volume at early time points after the insult, the combined results suggest that hypothermia exerts differential effects on the activation of microglia and astrocytes.

In the end, the balance between pro- and anti-inflammatory cytokines will determine the positive or negative influence of a neuroprotective agent. TGF-β1 is a neuroprotective compound and exogenous administration of TGF-β1 has been shown to decrease the infarct volume after stroke [48], but the spontaneous endogenous increase in TGF-β1 after stroke is not enough to cause effective neuroprotection [49]. However, hypothermia had no effect on the Et-1-induced increase in TGF-β1. Our study clearly demonstrates that hypothermia does not merely lead to a delay of events in the ischemic cascade. In contrast, specific up- or down-regulation of different cytokines occurs and these events take place at specific time points after hypothermia. Moreover, to optimize the application of hypothermia, the expression of genes (e.g. cytokines, chemokines, neurotrophic factors) and their role in ischemic damage or neuroprotection should be investigated.

In order to translate the optimal treatment protocol to the clinic, effects of hypothermia on peripheral immune suppression should also be taken into account. Immune suppression by either stroke or hypothermia may lead to an increased incidence of infections and a higher mortality [50]. However, the possibility that the inhibition of the immune response after stroke may be beneficial due to reduction of inflammatory responses in the brain should also be considered. Indeed, treatments with anti-inflammatory drugs have been shown to attenuate infarct size after experimental stroke [51]. Thus, combination of hypothermia with anti-infectious therapies may lead to less mortality and improved outcome in the hypothermic group in clinical settings.

## Conclusions

This study is the first to examine the neuroinflammatory response as a function of time after hypothermia in the Et-1 model for transient focal cerebral ischemia. Up to 1 week after the insult, hypothermia improved neurological outcome and significantly reduced infarct volume after the administration of Et-1. We suggest that hypothermia affects the early changes in the levels of pro-inflammatory cytokines after Et-1 administration. The pro-inflammatory IL-1β levels were reduced by hypothermic treatment, whereas those of TNF-α increased soon after the injection of Et-1. The current study is the first to show that phagocytic cells may play a dual role after stroke since the most prominent protection observed 3 days after the insult was associated with increased CD-68 expression. On the other hand, astrogliosis was reduced by the hypothermic treatment. Our data suggest that hypothermia modulates neuroinflammation after stroke, possibly allowing the therapeutic time window for other treatments (e.g. neuroprotective agents, anti-inflammatory drugs) to be extended after stroke.

## Competing interests

The authors declare that they have no competing interests.

## Authors' contributions

AGC carried out the experiments and drafted the manuscript, with the help of TZ. RK and SS helped to draft the manuscript and revised it critically. RK, SHI, SS and YM helped to conceive the design of the study and offered editorial assistance. All authors have read and approved the final version of the manuscript.
